# KetoFLEX 12/3 Diet and Cognitive Health: A Precision-Nutrition Perspective on Mechanisms, Emerging Evidence, and Future Directions

**DOI:** 10.3390/nu18132206

**Published:** 2026-07-07

**Authors:** Rammohan V. Rao, Kaavya G. Subramaniam, Julie Gregory, Aida L. Bredesen, Christine Coward, Sho Okada, Lance Kelly, Dale E. Bredesen

**Affiliations:** 1 Apollo Health, Burlingame, CA 94010, USA; 2 Department of Psychology, University of California, Davis, CA 95616, USA; 3 Pacific Neuroscience Institute, Santa Monica, CA 90404, USA

**Keywords:** Alzheimer’s disease, neurodegeneration, cognitive decline, memory, KetoFLEX 12/3, diet, multi-therapeutic program, precision medicine

## Abstract

Alzheimer’s disease (AD) is a multifactorial neurodegenerative disorder characterized by impaired glucose metabolism, mitochondrial dysfunction, inflammation, oxidative stress, and progressive cognitive decline. Because currently available pharmacological therapies provide only modest symptomatic benefit, nutrition-based interventions are increasingly being explored as complementary strategies for supporting brain metabolism and cognitive resilience. The KetoFLEX 12/3 dietary pattern, developed within the ReCODE (Reversal of Cognitive Decline) program, is a plant-rich, mildly ketogenic nutrition and lifestyle framework that integrates low-glycemic nutrition, time-restricted eating, and personalized metabolic optimization. The diet emphasizes deeply pigmented non-starchy vegetables, extra-virgin olive oil, nuts and seeds, omega-3-rich seafood, and minimally processed foods while limiting refined carbohydrates, sugars, processed foods, and selected grains and dairy products. Emerging mechanistic and clinical evidence suggests that KetoFLEX 12/3 may influence several pathways relevant to AD pathophysiology, including insulin signaling, mitochondrial bioenergetics, neuroinflammation, oxidative stress, autophagy, detoxification pathways, and gut–brain axis function. Observational findings from ReCODE-related studies have reported improvements in metabolic parameters, mood-related outcomes, cognitive measures, and brain volumetrics in participants adhering to multimodal precision-medicine interventions incorporating the KetoFLEX principles. Compared with traditional dietary models such as the Mediterranean or MIND diets, KetoFLEX 12/3 places greater emphasis on mild nutritional ketosis, meal timing, and metabolic personalization based on factors such as ApoE genotype and insulin sensitivity. The objective of this Perspective is to examine the mechanistic rationale, emerging evidence, limitations, and future research priorities for KetoFLEX 12/3 as a precision-nutrition framework for cognitive health in AD. Although much of the current evidence remains mechanistic, observational, or derived from multimodal intervention studies, the framework offers a biologically plausible precision-nutrition model that may inform future research and clinical investigation in cognitive decline.

## 1. Introduction

Alzheimer’s disease (AD) is a progressive neurodegenerative disorder and a major cause of morbidity, mortality, and loss of independence among older adults worldwide [1]. As global populations age, there is increasing urgency to develop strategies that not only delay cognitive decline but also support long-term brain health and resilience [2,3,4,5,6]. Despite decades of research, currently available pharmacological therapies provide modest clinical benefit for many patients, often slowing rather than reversing disease progression [7,8]. Recent biomarker-guided and disease-modifying approaches represent important advances, yet their variable outcomes underscore the complexity and heterogeneity of AD pathophysiology [7,8].

Several factors likely contribute to these therapeutic challenges. AD is characterized by a prolonged presymptomatic phase, whereas treatment is frequently initiated after substantial neuropathological changes have already occurred [9]. Increasing evidence further suggests that AD is not a single uniform disorder but rather a biologically heterogeneous syndrome involving multiple subtypes and interacting pathophysiological drivers [10]. Processes including inflammation, insulin resistance, vascular dysfunction, infections, toxin exposure, trophic insufficiency, and trauma may collectively contribute to disease initiation and progression [2,10,11,12].

While many therapeutic programs have focused primarily on amyloid-β and tau biology, increasing attention is being directed toward metabolic, inflammatory, vascular, and systems-level contributors that may shape disease expression and therapeutic response [7,8]. Against this background, multidomain approaches incorporating nutrition, lifestyle modification, and personalized metabolic optimization are increasingly being explored as complementary strategies for supporting cognitive health and addressing the biological complexity of AD [2,10,11].

Among the emerging approaches being explored within this broader precision-health paradigm is KetoFLEX 12/3, a plant-forward, mildly ketogenic dietary framework developed within the ReCODE (Reversal of Cognitive Decline) program. The framework combines principles of nutritional ketosis, time-restricted eating, and individualized metabolic optimization with the goal of supporting pathways implicated in cognitive decline. Interest in this approach has grown because it attempts to address several biological features commonly observed in AD, including impaired glucose metabolism, insulin resistance, inflammation, mitochondrial dysfunction, and gut–brain axis disturbances [2,10,11,13].

Importantly, KetoFLEX 12/3 is not presented here as a proven stand-alone treatment or replacement for established medical care. Rather, it is discussed as a hypothesis-generating, mechanistically informed precision-nutrition framework that warrants further controlled investigation.

### Scope of the Perspective

This article is presented as a Perspective rather than a systematic review or meta-analysis. The literature discussed was selected to illustrate mechanistic, clinical, and translational concepts relevant to KetoFLEX 12/3, cognitive health, and AD pathophysiology. Sources included peer-reviewed studies and reviews addressing nutrition and cognition, ketogenic metabolism, Mediterranean and MIND dietary patterns, insulin resistance, ApoE genotype, neuroinflammation, gut–brain signaling, fasting, and precision nutrition. Because this article is not a systematic evidence synthesis, no formal database search strategy, study-selection algorithm, or inclusion/exclusion criteria were applied. This limitation is acknowledged to reduce the risk of overstating the comprehensiveness of the evidence base.

## 2. Rationale for Nutrition-Based Interventions in Alzheimer’s Disease

Recent evidence from multiple research groups highlights substantial biological heterogeneity among individuals with AD, including differences in genetic, epigenetic, biochemical, metabolic, and inflammatory profiles that may influence therapeutic response [13,14,15,16,17,18,19]. These observations have stimulated increasing interest in multidomain and personalized approaches that address multiple contributors to disease expression simultaneously that are associated with improved cognitive and metabolic outcomes.

Within this framework, nutrition and lifestyle modification have emerged as important areas of investigation. Dietary patterns, specific food components, and targeted supplementation may influence pathways relevant to mild cognitive impairment (MCI) and AD, including insulin signaling, inflammation, oxidative stress, vascular function, and gut–brain interactions. Given the recognized associations among metabolic dysfunction, gut dysbiosis, obesity, diabetes, and neurodegeneration, dietary strategies that modulate these pathways have become central to contemporary dementia prevention and cognitive-health research [13,20,21,22,23]. Among the dietary models most extensively studied in cognitive aging are the Mediterranean diet, Dietary Approaches to Stop Hypertension (DASH), Mediterranean-DASH Intervention for Neurodegenerative Delay (MIND), and multidomain lifestyle programs such as the Finnish Geriatric Intervention Study to Prevent Cognitive Impairment and Disability (FINGER) [2,11,23,24,25,26,27,28,29,30,31]. These approaches generally emphasize plant-based foods rich in vegetables, fruits, nuts, legumes, whole grains, unsaturated fats, and antioxidant-rich food components, and have been associated with better cognitive performance, slower decline, or reduced dementia risk in multiple observational and interventional studies [32,33,34,35].

The FINGER trial provides a notable example of a multidomain strategy incorporating diet, physical activity, cognitive training, social engagement, and vascular risk management [4,5,36]. Although participants were cognitively unimpaired, they were at elevated dementia risk due to vascular factors, and the intervention group demonstrated significantly greater improvements across executive function, processing speed, and memory measures compared with controls. The DASH diet, for example, emphasizes foods low in sodium and rich in potassium, calcium, and magnesium [37], and adherence to this and related dietary frameworks has been associated with improved cognitive outcomes, likely mediated through vascular risk reduction [32,33,34,35]. Building on these foundations, the MIND diet, specifically designed to target neurodegeneration has demonstrated more consistent associations with preserved cognition and reduced Alzheimer’s disease risk across multiple cohorts [25,35].

Beyond whole dietary patterns, several dietary components commonly emphasized in these approaches including vegetables, fruits, legumes, whole grains, nuts, and olive oil, have demonstrated independent associations with cognitive health. Bioactive constituents such as unsaturated fatty acids, antioxidants, and dietary flavonoids may further support neuroprotection and attenuate cognitive decline [35,38,39,40,41,42,43,44,45,46]. These observations have fueled growing interest in precision-nutrition frameworks that tailor dietary interventions to individual metabolic, genetic, and clinical profile. It is important to emphasize that non-ketogenic dietary patterns, particularly Mediterranean and MIND diets, have among the strongest epidemiological and clinical support for cognitive health. These diets are associated with slower cognitive decline and reduced AD risk, likely through vascular, anti-inflammatory, antioxidant, and metabolic mechanisms. Similarly, extra-virgin olive oil and other unsaturated fat sources that are recommended as part of these structured diets may provide neuroprotective benefits independent of ketosis.

Similarly, KetoFLEX 12/3 represents one such emerging approach. KetoFLEX 12/3 should not be interpreted as superior to the above-mentioned dietary models based on current evidence, but rather as a related precision-nutrition framework that incorporates several Mediterranean-like features while adding meal timing, carbohydrate modulation, and mild nutritional ketosis for selected metabolic contexts. Developed within the ReCODE (Reversal of Cognitive Decline) program, it is a plant-rich, mildly ketogenic nutritional program that includes time-restricted eating and precision personalization based on genetics (e.g., ApoE4 status) and biochemical profiles [2,11,13,47]. The following sections examine the mechanistic rationale, emerging evidence base, and translational considerations underlying this framework in the context of cognitive health and AD.

## 3. Gut–Brain Axis Vulnerability and Dietary Components That Challenge Intestinal Integrity and Cognitive Resilience

Disturbances of the gut–brain axis including dysbiosis, altered intestinal permeability, and microbial translocation are increasingly being investigated as contributors to neuroinflammation and cognitive decline. Individuals with AD frequently exhibit elevated lipopolysaccharide (LPS), altered zonulin signaling, and shifts in commensal microbial populations, changes that may increase systemic inflammatory burden and have been associated with neuronal dysfunction, impaired synaptic plasticity, and progression of AD pathology [48,49,50,51,52].

Certain dietary components may influence these pathways in susceptible individuals [53,54,55]. Gluten and gliadin, for example, have been shown to modulate intestinal permeability through zonulin-related mechanisms in genetically or immunologically vulnerable populations and may contribute to gastrointestinal or inflammatory responses in selected individuals [56,57,58]. Although whole grains are generally considered health-promoting and non-inflammatory in healthy populations, these effects may become more clinically relevant in individuals exhibiting pre-existing gut vulnerability, metabolic dysfunction, or altered immune responsiveness [59,60,61].

Simple carbohydrates that are processed or ultra-refined foods (sugary beverages, chips, cookies, pastries, cereals, breads, crackers, pastas, and most desserts) may impair gut microbial diversity, promote pro-inflammatory microbiota, disrupt gut barrier integrity, and contribute to metabolic and inflammatory dysregulation. These foods can raise blood glucose levels as rapidly and significantly as added sugar, triggering metabolic disturbances and promoting inflammation, especially when combined with the refined, industrially engineered seed oils common in many processed products [62,63,64,65,66,67]. In contrast, carbohydrate sources that deliver fiber, resistant starch, and minimal processing tend to support microbial diversity and gut health [68,69].

Sugars in all its forms including high-fructose corn syrup, brown rice syrup, barley malt, dextrose, sucrose, fructose and lactose among others are pervasive in ultra-processed foods and contribute to chronic elevations in postprandial glucose, HbA1c, and fasting insulin, making their reduction a high-priority dietary intervention [70,71,72,73,74,75]. Beyond overtly sweetened foods, added sugars are present in most savory processed products thereby complicating glycemic control even in individuals who do not perceive their diet as high in sugar. Excessive sugar exposure promotes insulin resistance, advanced glycation end-product (AGE) formation, oxidative stress, and neuroinflammation, all of which impair cerebral glucose metabolism and synaptic function and are increasingly implicated in cognitive decline and AD pathophysiology [75,76,77]. Adopting a whole-foods dietary pattern makes it easier to avoid these hidden sources, and many individuals experience a natural reduction in sugar cravings over time [78].

As mentioned above, excess intake of sugars, simple carbohydrates, and refined grains plays a significant role in the global rise of obesity, pre-diabetes, and type 2 diabetes [70,71,72,73,74]. These metabolic disturbances impair the brain’s ability to efficiently utilize glucose, its primary fuel and can also hinder the transition into ketosis which is a critical alternative energy pathway for neuronal function [74,76,79,80]. Research studies have linked insulin resistance and chronic hyperglycemia (which may be accompanied by reactive hypoglyemia) to neurodegeneration, contributing to the concept of AD as ‘type 3 diabetes’, while simultaneously showing that insulin resistance drives systemic inflammation, thereby compounding both metabolic and neurological risk [77,81,82].

In addition, most dairy products, particularly those rich in whey proteins, produce a disproportionately high insulin response relative to their carbohydrate content [83]. While typically well tolerated in metabolically healthy individuals, this insulinogenic effect may complicate metabolic goals in selected populations characterized by insulin resistance or cognitive impairment [84,85]. In addition, dairy-associated immune reactivity has been reported in some patient subgroups, and ApoE4 carriers may demonstrate altered inflammatory or lipid responses to certain saturated fats [86,87,88,89,90,91].

Emerging evidence further suggests that not all dairy exerts identical physiological effects. Conventional dairy containing predominantly A1 β-casein has been associated in some studies with greater gastrointestinal discomfort, inflammatory signaling, oxidative stress, and altered gut microbial patterns, potentially mediated through generation of the peptide β-casomorphin-7 (BCM-7) [86,92,93,94,95]. By contrast, A2 β-casein dairy appears to exhibit improved digestive tolerance and lower inflammatory responses in some populations, with preliminary findings suggesting possible differences in gut–brain interactions and cognitive outcomes [86,92,93,94,95]. Accordingly, within individualized dietary frameworks such as KetoFLEX 12/3, selective reduction in conventional dairy with allowance for small amounts of A2 dairy in those who tolerate it may represent one pragmatic strategy for addressing metabolic and gastrointestinal variability.

Since approximately 50–60% of individuals with AD carry at least one ApoE4 allele, and emerging evidence suggests ApoE4 may influence lipid metabolism, glucose regulation, and inflammatory responses, dietary strategies that reduce refined carbohydrates and support metabolic flexibility have attracted increasing interest in this population [96,97,98,99].

## 4. Alzheimer’s Disease, Metabolic Dysfunction and Ketosis

A defining feature of AD is impaired cerebral glucose utilization, particularly in temporoparietal (often including posterior cingulate and precuneus) and prefrontal regions. Neuroimaging studies consistently demonstrate reduced cerebral glucose uptake decades before the onset of clinical symptoms. This hypometabolic phenotype is especially pronounced in ApoE4 carriers, in whom reductions in cerebral glucose metabolism have been observed as early as the third decade of life, well before detectable cognitive impairment, suggesting a genetically mediated vulnerability in neuronal energy utilization [100,101]. This early hypometabolism is tightly linked to systemic and cerebral metabolic dysfunction, including insulin resistance, chronic hyperglycemia, impaired mitochondrial function, and neuroinflammation. These overlapping metabolic disturbances not only reduce neuronal glucose availability but also compromise synaptic signaling and accelerate amyloidogenic and tau-related pathways [76,77,102,103,104]. Importantly, ketone uptake and utilization remain relatively intact even in advanced stages of AD, making ketosis a promising metabolic strategy to circumvent defective insulin signaling and support brain energetics. Nutritional interventions aimed at improving glucose regulation and enhancing alternative cerebral fuel availability including low-glycemic diets, time-restricted eating, and ketogenic strategies that increase ketone availability have been associated with improvements in cognition, executive function, and memory in individuals with MCI and early AD. These benefits likely stem from ketones’ ability to enhance mitochondrial efficiency, reduce oxidative stress, modulate inflammation, and restore metabolic flexibility [105,106,107,108,109,110,111,112,113,114,115]. Collectively, these findings support continued investigation of dietary approaches such as KetoFLEX 12/3, which seek to address metabolic dysfunction and support neuronal energy metabolism through mild, physiologic nutritional ketosis within a metabolically adaptive framework.

## 5. KetoFLEX 12/3 Diet-A Precision-Nutrition Model Tailored to Neurodegenerative Vulnerability

**Figure 1:**
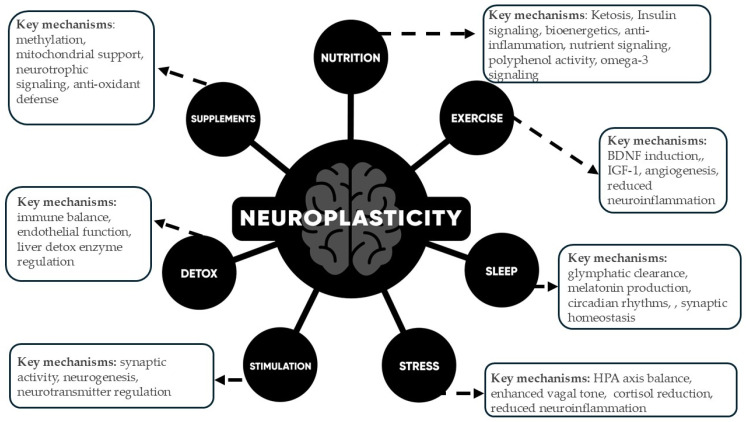
Mechanistic framework of the KetoFLEX 12/3 approach in Alzheimer’s Disease and Cognitive Health. Proposed mechanistic framework illustrating how the multidomain components of the KetoFLEX 12/3 program may converge on biological pathways relevant to neuroplasticity and cognitive resilience in Alzheimer’s disease. Nutritional composition, intermittent fasting/meal timing, physical activity, sleep optimization, stress regulation, cognitive stimulation, detoxification strategies, and targeted supplementation are proposed to influence interconnected processes including metabolic flexibility, mitochondrial energetics, inflammation, vascular function, synaptic signaling, and neurotrophic support. The figure is intended as a conceptual representation of mechanisms discussed throughout this article. Abbreviations: BDNF, brain-derived neurotrophic factor; IGF-1, insulin-like growth factor 1; HPA, hypothalamic-pituitary-adrenal (axis).

The KetoFLEX 12/3 diet integrates ketogenic principles with a precision-medicine framework [2,11,13]. Unlike population-level dietary programs such as FINGER, DASH, or MIND, KetoFLEX 12/3 applies a precision medicine framework to individualize interventions across seven foundational lifestyle domains, with the goal of supporting neuroplasticity, restoring systemic balance, and improving cognitive function (Figure 1) [2,11,13].

Since AD is preceded and accompanied by the brain’s inability to effectively use glucose as fuel the “keto” in KetoFLEX 12/3 refers to ketosis, the process by which the body uses dietary fat and breaks down stored body fat to use as fuel. The “flex” refers to metabolic flexibility, the capacity to efficiently transition between glucose-and fat-derived fuels in response to nutritional and energetic demands [113,116]. The therapeutic intent of KetoFLEX 12/3 is therefore not the promotion of chronic ketosis, but rather the restoration of insulin sensitivity and metabolic adaptability, enabling the brain to better utilize glucose while also accessing ketones as a complementary fuel source. Within the KetoFLEX framework, dietary composition, meal timing, and physical activity are intended to periodically promote mild physiological ketosis, typically reflected by measurable increases in circulating ketone bodies, while maintaining overall metabolic flexibility and nutritional adequacy [109,117,118].

The rationale for this approach derives from evidence suggesting that carbohydrate tolerance, glycemic regulation, and metabolic flexibility may differ substantially across populations. Although grains are widely recognized as nutrient-dense foods with established health benefits in general populations, their carbohydrate content may contribute to post-prandial hyperglycemia, insulin resistance, glycotoxicity, impaired cerebral glucose utilization, gut permeability, and dysbiosis in selected individuals characterized by insulin resistance, impaired glucose metabolism, or ApoE4-associated metabolic sensitivity [102,112,119,120]. Within this context, KetoFLEX 12/3 is structured to emphasize glycemic stability, mild nutritional ketosis, and individualized metabolic responsiveness [109,117,118].

KetoFLEX 12/3 may therefore be viewed as a mechanistically informed precision-nutrition approach. Its dietary structure is designed to support several interconnected goals: enhancing ketone availability, moderating glycemic variability and insulin exposure, supporting gut barrier integrity, and attenuating pathways linked to neuroinflammation and metabolic dysfunction [121,122,123,124,125]. The selective limitation of grains, legumes, and some dairy products is framed within this review not as a universal recommendation, but as a personalized nutritional strategy that may be relevant for specific metabolic or neurobiological contexts encountered in individuals with MCI or early AD [126,127,128,129,130,131].

Thus, KetoFLEX 12/3 may be viewed as complementary to, rather than competitive with, Mediterranean and MIND dietary frameworks. All three approaches emphasize nutrient density, plant-rich eating patterns, and reduction in ultra-processed foods, with KetoFLEX 12/3 placing additional emphasis on meal timing, metabolic flexibility, and individualized adaptation based on metabolic and genetic characteristics.

### 5.1. Metabolic Dysfunction as an Early Driver of AD and the KetoFLEX 12/3 Diet

Neuroimaging and metabolomic studies consistently show that cerebral glucose hypometabolism precedes clinical symptoms of AD by years to decades. This “type 3 diabetes” phenotype reflects brain insulin resistance and impaired mitochondrial oxidation, ultimately contributing to energetic failure and synaptic dysfunction [77,132,133]. Restoring metabolic flexibility, the ability to efficiently utilize glucose, fatty acids, or ketones, is therefore essential for maintaining cognitive function [122,126,134,135].

The KetoFLEX 12/3 dietary pattern is designed to promote mild nutritional ketosis and may increase circulating β-hydroxybutyrate, a preferred neuronal substrate that bypasses impaired glucose pathways and enhances mitochondrial ATP production [136,137,138]. Beyond serving as an alternative fuel, ketones also function as signaling molecules: they inhibit histone deacetylases, promote expression of brain-derived neurotrophic factor (BDNF), and reduce neuroinflammation [139,140]. These combined effects support synaptic plasticity, mitochondrial resilience, and neuronal survival, core domains compromised early in AD pathogenesis (Figure 1).

### 5.2. Inflammation, Oxidative Stress, and Vascular Dysfunction

Systemic inflammation and oxidative stress accelerate neuronal injury through activation of NF-κB signaling, microglial overactivation, and vascular endothelial dysfunction [141,142,143]. Dietary patterns rich in polyphenols, omega-3 fatty acids, and antioxidants, particularly extra-virgin olive oil, leafy greens, mushrooms, herbs, and spices can attenuate these inflammatory and oxidative processes [144,145,146]. Although Mediterranean and MIND dietary patterns are consistently associated with improvements in metabolic health, they do not specifically emphasize nutritional ketosis or deliberate promotion of metabolic flexibility as therapeutic targets, particularly in individuals with insulin resistance or ApoE4-associated vulnerability. KetoFLEX 12/3 was developed to address this gap by restoring metabolic flexibility through strategic carbohydrate modulation, enabling regular periods of physiologic endogenous ketosis while maintaining the anti-inflammatory benefits of plant-based nutrition [121,147,148,149,150].

### 5.3. Fasting and Circadian Metabolism

Intermittent fasting and time-restricted feeding support autophagy, promote mitochondrial biogenesis, and improve insulin sensitivity, mechanisms essential for neuronal maintenance and metabolic health [151,152,153]. The “12/3” structure of KetoFLEX which requires fasting for ≥12 h daily with the final meal consumed ≥3 h before bedtime, leverages circadian rhythms to reduce post-prandial glycemia, enhance sleep quality, and synchronize metabolic and neurophysiological processes [154]. Human studies demonstrate that even modest fasting windows can elevate ketone levels, reduce systemic inflammation, and improve cognitive performance [152,155,156]. In selected individuals particularly ApoE4 carriers, longer fasting intervals may be potentially beneficial, reflecting enhanced reliance on lipid- and ketone-based fuel utilization [105,121]. Such approaches are implemented cautiously and individualized within the KetoFLEX framework, with attention to body composition, strength, glycemic control, and overall physiologic resilience.

## 6. Core Food Components of the KetoFLEX 12/3 Diet

Cognition-supportive dietary patterns such as the Mediterranean, FINGER, DASH, and MIND diets consistently promote whole grains and permit low to moderate dairy intake, while limiting refined or simple carbohydrates [23,24,25,26,27,28,29,30,31]. This stands in contrast to the KetoFLEX 12/3 therapeutic framework, which replaces grains with non-starchy, deeply pigmented plant foods with a low glycemic impact. These foods span diverse colors and botanical families, maximizing exposure to polyphenols, carotenoids, vitamins, minerals, and other bioactive compounds that support metabolic, vascular, and cognitive resilience [25,157,158].

### 6.1. Vegetables and Fruits

Leafy green vegetables including kale, spinach, chard, arugula, beet greens, collards, and watercress form the foundation of the plant component of KetoFLEX 12/3. These foods provide folate, vitamin K, magnesium, and nitrates, and are associated with homocysteine reduction, improved endothelial function, and nitric oxide-mediated vascular support relevant to brain health [25,159,160,161,162].

Cruciferous vegetables (e.g., broccoli, Brussels sprouts, cauliflower, bok choy, rapini), together with allium vegetables (garlic, onions, leeks, shallots), are emphasized for their glucosinolate and organosulfur-derived bioactive compounds. Preparation methods involving chopping and holding prior to cooking and gentle heat exposure help preserve myrosinase activity and support sulforaphane formation [163,164,165,166].

Fruit intake within KetoFLEX 12/3 is selective, favoring low-glycemic, high-phytonutrient options. Wild or minimally sweet berries including blueberries, strawberries, raspberries, blackberries, cranberries, currants, bilberries, and pomegranate are prioritized for their polyphenolic density and neuroprotective potential [167,168,169]. Tart cherries are associated with improved metabolic regulation, reduced oxidative stress, and memory-related outcomes [170,171], while citrus fruits such as lemons and limes are included for flavor and vitamin C content with minimal glycemic burden [172]. In contrast, modern selectively bred, high-sugar fruits are deemphasized, and tropical fruits with high glycemic load are less preferred. Small quantities of low-glycemic unripe fruits are considered compatible in metabolically favorable contexts [173,174,175].

For clarity, while foods such as tomatoes, olives and avocados are botanically classified as fruits, they are mentioned within this category based on their low glycemic impact, metabolic effects and culinary use. Avocados are included as multifunctional foods supplying monounsaturated fatty acids, fiber, potassium, and magnesium, while also facilitating nutritional ketosis [154,162,176]. Olives and olive-derived products provide monounsaturated fats and polyphenols with anti-inflammatory and antimicrobial properties with brine-fermented varieties additionally contributing to microbial diversity [177,178]. Tomatoes contribute flavonoids, phenolic compounds, and carotenoids including lycopene and β-carotene, whose bioavailability is enhanced when consumed with dietary fat [161,179,180].

Culinary mushrooms, particularly *Hericium erinaceus* (Lion’s Mane), though taxonomically distinct from plants, are included within this category based on their functional nutritional properties. Mushrooms provide a range of bioactive compounds including β-glucans, ergothioneine, erinacines, hericenones, selenium, and other polysaccharides with documented antioxidant, immunomodulatory, and neuroprotective potential [181,182]. In vivo studies suggest that supplementation with medicinal and culinary mushrooms (e.g., Lion’s Mane, reishi, cordyceps) may support neurotrophic signaling and cognitive function. In addition, observational studies of commonly consumed mushroom varieties have reported associations with a reduced risk of cognitive impairment in aging populations [181,182,183,184].

### 6.2. Herbs, Spices, Teas, and Coffee

Spices, long used as culinary agents, are increasingly recognized for their neuroprotective potential in aging and dementia. Bioactive compounds derived from commonly used spices including turmeric, members of the pepper family, saffron, ginger and cinnamon, modulate multiple pathological processes relevant to Alzheimer’s disease by suppressing neuroinflammation, reducing oxidative stress, inhibiting acetylcholinesterase activity, and limiting amyloid-β and tau aggregation. Several also influence mitochondrial function, synaptic plasticity, and intracellular signaling pathways such as NF-κB, Nrf2, and MAPK that govern neuronal survival [185,186,187].

Teas, particularly green tea and matcha, are valued for epigallocatechin-3-gallate (EGCG), a flavanol with demonstrated neuroprotective and metabolic effects [188,189,190]. Brewing techniques that minimize excessive heat and microplastic exposure are favored [191,192].

Coffee derived from Coffea arabica is recognized as a major dietary source of antioxidants with potential metabolic and cognitive relevance [193,194,195]. While a variety of coffee forms are increasingly consumed, preparation methods can influence polyphenol content, diterpene levels, and glycemic impact [196,197]. The potential cognitive and metabolic benefits of coffee are primarily attributed to its antioxidant and anti-inflammatory constituents [198]. Within the KetoFLEX framework, coffee is best consumed without added sugars or sweetened creamers to avoid counteracting metabolic benefits.

### 6.3. Healthy Fats and Oils

Healthy fats serve as metabolic and structural cornerstones of the KetoFLEX 12/3 pattern, supporting nutritional ketosis, mitochondrial function, neuronal membranes, and myelin integrity [74,102]. The framework distinguishes monounsaturated fats (e.g., avocados, olives, olive oil, nuts), polyunsaturated fats including omega-3 fatty acids from marine and plant sources and omega-6 fatty acids from whole foods and saturated fats derived primarily from minimally processed sources [199,200,201].

Industrial trans fats and refined seed oils are excluded because of their adverse effects on lipid metabolism, inflammation, and cardiometabolic risk. KetoFLEX 12/3 aims to correct the excess omega-6 exposure typical of Western dietary patterns by reducing industrial seed oils and increasing marine- and plant-derived omega-3 intake [202,203,204].

High-polyphenol extra-virgin olive oil is a central component of the KetoFLEX 12/3 framework because its monounsaturated fat and polyphenol profile has been associated with improved insulin sensitivity, healthier lipid metabolism, reduced inflammation, and activation of neuroprotective pathways relevant to mitochondrial function, autophagy, amyloid and tau biology, and brain-derived neurotrophic factor (BDNF) signaling [205,206,207].

### 6.4. Nuts and Seeds

Nuts and seeds are incorporated as nutrient-dense sources of unsaturated fats, dietary fiber, minerals, and diverse phytonutrients [208]. In individuals with ApoE4-associated lipid sensitivity or cholesterol hyperabsorption, greater emphasis is placed on monounsaturated (MUFA) and polyunsaturated fatty acids (PUFA) rather than saturated fats [208,209,210]. Accordingly, the KetoFLEX dietary patterns favor walnuts, macadamias, hazelnuts, pecans, pistachios, almonds, flax, and chia seeds. Preparation techniques such as soaking and sprouting have been shown to reduce antinutritional factors including phytates, lectins, and enzyme inhibitors, thereby improving mineral bioavailability and digestive tolerance [211,212,213]. Peanuts are relatively de-emphasized because of concerns regarding susceptibility to mold contamination and aflatoxin exposure [214]. Flax and chia seeds are particularly rich in plant-derived omega-3 fatty acids (α-linolenic acid, ALA), macadamia nuts provide predominantly MUFAs, walnuts are enriched in PUFAs, and almonds, hazelnuts, pistachios, and pecans offer favorable blends of MUFA and PUFA associated with cardiometabolic and cognitive health [215,216].

### 6.5. Prebiotics, Probiotics and Resistant Starch

Within KetoFLEX 12/3, targeted use of prebiotics, probiotics, and resistant starch is encouraged to support microbial diversity, strengthen gut barrier function, and favorably influence metabolic and neuroinflammatory pathways. Prebiotic fibers are non-digestible carbohydrates selectively fermented by beneficial colonic bacteria that are recognized for their role in short-chain fatty acid (SCFA) production, particularly butyrate, which supports gut barrier integrity and may contribute indirectly to ketone availability. Many non-starchy vegetables, roots, and tubers provide these substrates [217,218,219].

Probiotic foods derived from traditional fermentation are included as natural sources of Lactobacillus, Bifidobacterium, and, in the case of natto, Bacillus subtilis. Preference is given to non-pasteurized, non-vinegar-preserved, and low-sugar preparations, with recognition that individual tolerance may vary, particularly in those with increased gut permeability. Short-term probiotic supplementation is viewed as adjunctive, with long-term emphasis placed on food-based microbial exposure [220,221,222].

Resistant starch represents a distinct class of carbohydrates that behaves physiologically like fiber, escaping small intestinal digestion and undergoing fermentation in the colon. Resulting SCFAs are associated with improved insulin sensitivity, lipid metabolism, satiety signaling, fat oxidation, and gut health. However, because it can increase glucose exposure in some individuals, it is typically introduced in moderation and after insulin resistance has improved. Sources include legumes (when appropriately prepared), tubers such as colored potatoes when cooked and cooled, and certain unripe fruits such as green plantains or bananas [223,224,225,226].

In addition to resistant starch and fermented foods, polyphenol-rich foods including berries, olive oil, herbs, spices, tea, cocoa, and colorful vegetables may favorably influence microbial diversity and short-chain fatty acid production. These effects have been associated with enhanced gut barrier integrity, reduced systemic inflammation, and modulation of gut-derived signaling pathways increasingly implicated in neurodegenerative disorders.

### 6.6. Meat, Seafood and Poultry

KetoFLEX distinguishes minimally processed, pasture-raised or wild-sourced animal foods from industrially produced animal products. Grass-fed, pasture-raised, hormone and antibiotic-free animal foods are preferred to reduce exposure to environmental toxins stored in adipose tissue [227,228]. Red meat intake is moderated, in part due to Neu5Gc content, while venison is avoided because of concerns related to chronic wasting disease [229,230,231].

Protein intake within KetoFLEX 12/3 is considered through a metabolic and longevity paradigm, with emphasis on achieving nutritional adequacy while avoiding chronic overconsumption. In selected metabolic contexts, short periods of modest protein reduction may be incorporated to support pathways related to autophagy and metabolic flexibility, while maintaining muscle mass and overall nutritional sufficiency. Plant foods are recognized as important contributors to total protein intake, and periodic moderation of animal protein consumption is viewed as compatible with long-term metabolic, healthy-aging, and cognitive objectives [232,233,234].

Seafood, particularly wild-caught cold-water species summarized by the “SMASH” acronym (salmon, mackerel, anchovies, sardines, herring), is prioritized for its EPA/DHA content and relatively low mercury burden. Carefully certified farmed seafood is considered acceptable when appropriately vetted. Shellfish and mollusks are included when sourced from low-contaminant environments [74,235].

For poultry, KetoFLEX 12/3 conceptually favors truly pastured birds with access to forage and minimal reliance on supplemental grain. The framework highlights limitations of common labeling terms such as “cage-free” or “free-range,” noting that even organic poultry typically receives grain-based feed. Fully pastured poultry, when available, provides a more favorable omega-3 profile [203]. Pasture-raised eggs are emphasized for their content of omega-3 fatty acids, vitamin B12, folate, fat-soluble vitamins, and choline, with egg yolks supporting synaptic membrane integrity and neurotransmission [236,237].

### 6.7. Sweeteners and Cocoa Flavanols

A core objective of KetoFLEX 12/3 is recalibration of sweet taste with a reduced reliance on sweetness as a marker of metabolic adaptation away from ultra-processed foods [238,239]. Non-nutritive sweeteners are generally minimized, as even non-caloric sweetness may influence insulin signaling and the gut microbiome [66,240,241,242]. When used selectively, certain low-glycemic sweeteners such as pure stevia, monk fruit extract or allulose are considered more compatible [243,244,245,246,247,248,249,250,251,252].

In metabolically flexible individuals, limited use of natural sweeteners such as honey or maple syrup may be incorporated in nutritionally dense meal contexts. Sugar alcohols are generally discouraged due to gastrointestinal intolerance and potential microbiome effects [253,254,255].

Cocoa flavanols are of interest because of reported vascular and neurocognitive effects; however, concerns regarding sugar content and heavy metal contamination necessitate careful selection. High-cacao dark chocolate with minimal sugar and verified low levels of cadmium and lead is preferred and consumed sparingly [256,257,258,259,260].

## 7. Precision and Personalization

Given proposed models of AD heterogeneity including Inflammatory (type 1), Atrophic (type 2), Glycotoxic (type 1.5), Toxic (type 3), Vascular (type 4) and Traumatic (type 5), a uniform dietary strategy may not adequately address the biological variability observed across individuals with cognitive decline [2,15,77,261]. The KetoFLEX 12/3 framework is designed to be adaptable, allowing dietary composition, fasting duration, and food selection to be adjusted according to variables such as ApoE genotype, body composition, insulin sensitivity, gastrointestinal status, hormonal milieu, and coexisting clinical conditions. For example, individuals with insulin resistance may prioritize glycemic reduction, carbohydrate modulation, and fasting optimization to improve metabolic flexibility. In ApoE4 carriers, some implementations of KetoFLEX 12/3 place greater emphasis on polyphenol-rich unsaturated fats, non-starchy plant foods, and moderation of saturated fat exposure; however, these adaptations are based on emerging evidence regarding ApoE-associated differences in lipid metabolism, glucose regulation, and inflammatory responses and should be regarded as individualized considerations rather than established clinical recommendations. Longer fasting windows (up to 16 h) may be considered only cautiously and in selected individuals, with attention to age, body composition, frailty risk, glycemic stability, sleep, medication use, and overall clinical context. Rather than advocating a uniform dietary prescription, this approach reflects the broader premise that nutritional requirements and therapeutic responsiveness may differ across biologically heterogeneous AD populations [97,121,152,262].

Although the term KetoFLEX 12/3 was not explicitly used in several earlier precision-medicine studies of cognitive decline, many of these interventions incorporated core elements that later became formalized within the framework, including low-glycemic nutrition, mild nutritional ketosis, time-restricted eating, metabolic optimization, and individualized treatment strategies. Improvements reported in these multimodal studies provided part of the clinical rationale for the development and refinement of KetoFLEX 12/3 [2,10,11,13,47,263]. Thus, KetoFLEX 12/3 evolved from clinical precision-medicine programs that had already demonstrated feasibility and cognitive benefit, rather than being derived solely from theoretical or mechanistic considerations.

Nevertheless, the independent contribution of the dietary framework remains difficult to isolate within multifactorial programs, underscoring the need for future biomarker-guided randomized controlled trials specifically evaluating KetoFLEX 12/3. Accordingly, KetoFLEX 12/3 may be viewed as an emerging precision-nutrition framework and a useful conceptual model for future investigations examining personalized dietary strategies in cognitive decline and Alzheimer’s disease [97,121,152,262].

However, practical implementation remains an important consideration. Older adults may face challenges related to meal timing, dietary preferences, medication schedules, frailty, caregiver support, and long-term adherence. Accordingly, KetoFLEX 12/3 is intended to be implemented progressively and individualized according to nutritional status, body composition, comorbidities, and overall clinical context rather than applied as a rigid dietary prescription.

## 8. Conclusions

Alzheimer’s disease is increasingly recognized as a biologically heterogeneous disorder involving disturbances in metabolism, inflammation, vascular function, mitochondrial energetics, and gut–brain signaling. Within this evolving landscape, nutrition-based interventions are receiving growing attention as complementary components of multidomain strategies for cognitive health. KetoFLEX 12/3 represents an emerging precision-nutrition framework that combines plant-forward, mildly ketogenic nutrition, meal timing, and individualized metabolic tailoring to address pathways relevant to cerebral glucose dysregulation, neuroinflammation, oxidative stress, and metabolic resilience. Rather than functioning as a universal dietary prescription, the framework is intended to accommodate biological variability across individuals with cognitive decline.

At present, evidence specific to KetoFLEX 12/3 remains limited and derives largely from mechanistic studies, observational findings, multimodal intervention programs, and related ketogenic or metabolic literature rather than large randomized trials evaluating the dietary framework in isolation. While observational studies, multimodal intervention programs, and emerging translational data provide encouraging signals, important questions remain regarding long-term adherence, implementation feasibility, biomarker-guided personalization, and validation through larger controlled studies. Future investigations incorporating metabolic phenotyping, ApoE genotype stratification, gut–brain biomarkers, and precision-nutrition trial designs will be essential for clarifying which individuals are most likely to benefit from individualized dietary strategies in Alzheimer’s disease and related cognitive disorders.

## Data Availability

Not Applicable.
